# Serum Hepcidin and GDF-15 levels as prognostic markers in urothelial carcinoma of the upper urinary tract and renal cell carcinoma

**DOI:** 10.1186/s12885-019-5278-0

**Published:** 2019-01-15

**Authors:** Lisa Traeger, Ines Ellermann, Helene Wiethoff, Janina Ihbe, Inka Gallitz, Maria Eveslage, Rudolf Moritz, Edwin Herrmann, Andres Jan Schrader, Andrea U. Steinbicker

**Affiliations:** 1 Department of Anesthesiology, Intensive Care and Pain Medicine, University Hospital Muenster, University of Muenster, Albert-Schweitzer Campus 1, Building A1, 48149 Muenster, Germany; 2 Department of Pharmacy, University Hospital Muenster, University of Muenster, Muenster, Germany; 3 Institute of Biostatistics and Clinical Research, University Hospital Muenster, University of Muenster, Münster, Germany; 4 Department of Urology, University Hospital Muenster, University of Muenster, Muenster, Germany; 5 Present Address: Department of Urology, St. Antonius Hospital, Gronau, Germany; 6 Present Address: Department of Urology, Prosper Hospital, Recklinghausen, Germany

**Keywords:** Hepcidin, GDF-15, Prognostic markers, Urothelial carcinoma of the upper urinary tract, Renal cell carcinoma

## Abstract

**Background:**

Cancer is a life-threatening disease that causes every fourth death. It is often hard to determine the time point of progression. Therefore, biomarkers for cancer entities that indicate disease progression or aggressiveness and thereby guide therapeutic decisions are required. Unfortunately, reliable biomarkers are rare. In this study, the potential of serum hepcidin and serum GDF-15 as biomarkers that correlate with patient’s survival in the two entities upper urinary tract urothelial carcinomas (UUTUC) and renal cell carcinoma (RCC) were analyzed.

**Methods:**

In this retrospective study *n* = 38 patients suffering from UUTUC, *n* = 94 patients suffering from RCC and *n* = 21 patients without infections or cancer, all hospitalized at the University Hospital Muenster, were included. Serum samples of patients were retrospectively analyzed. Serum hepcidin and GDF-15 levels were measured and correlated to aggressiveness and progression of the disease as well as patient’s outcome.

**Results:**

For both entities, UUTUC and RCC, serum hepcidin levels as well as serum GDF-15 levels were increased compared to sera of controls. High serum hepcidin and GDF-15 levels were associated with metastases and cancer relapse. Also, in both entities, the overall survival was decreased in patients with increased serum hepcidin and GDF-15 levels. Hence, high serum hepcidin and GDF-15 levels correlated with patient’s outcome.

**Conclusion:**

To conclude, the data of this study show a correlation of high serum hepcidin and GDF-15 levels with aggressiveness and progression of the disease and demonstrate potential prognostic properties of serum hepcidin and GDF-15 levels. The data support the further assessment of serum hepcidin and GDF-15 as prognostic markers in RCC and UUTUC.

## Background

About 25 % of the European population dies due to cancer. After diagnosis, treatment decisions are sometimes hard to find. While some cancers progress fast and are aggressive per se (such as lung cancer), others progress slowly and can be treated well (such as prostate cancer (PCa)). It is difficult to foresee the time point at which progression occurs and to decide on treatment. Biomarkers that indicate disease progression or aggressiveness may help in these situations. Unfortunately, reliable biomarkers are rare. The hepatic hormone hepcidin has recently gained more attention in various cancer entities such as PCa and breast cancer [1–3]. Hepcidin is known as the main regulator of iron homeostasis that causes the internalization and degradation of the sole known iron exporter ferroportin [4]. The reduction of ferroportin on the cell membrane prevents iron absorption in the intestine as well as iron release from hepatocytes, enterocytes and macrophages into the circulation and thereby decreases iron availability [5]. The expression of hepcidin is induced by the BMP signaling pathway as well as different cytokines that are released in inflammatory settings. If hepcidin is induced for a prolonged period, the iron restriction leads to the development of the anemia of chronic disease (ACD), which is also named anemia of inflammation (AI) [6]. In addition, estrogen and testosterone regulate hepcidin [7, 8].

Hepcidin is typically produced in the liver, but cancer cells may be able to produce hepcidin themselves. For cancer under hormonal regulation, such as breast cancer and PCa, an increase in hepcidin expression in cancer tissue was observed. This expression profile correlated with tumor growth and metastasis [1, 3].

Also, increased serum hepcidin levels were associated with metastasized disease and poor outcome in prostate, breast, colon and non-small cell lung cancer as well as renal cell carcinoma and non-Hodgkin lymphoma [1–3, 9–12]. The increase in serum hepcidin levels leads to reduced ferroportin levels. Ferroportin are the iron absorption channels expressed on enterocytes, hepatocytes, macrophages and cancer cells [13]. This fact leads to the development of anemia, and anemia increases patient’s morbidity and mortality.

Expression of Growth/differentiation factor 15 (GDF-15), a member of the TGF-β family that plays a role in tissue regeneration and healing, is greatly enhanced in various types of cancer [2, 14]. A cancer-associated increase in GDF-15 levels was suggested to have a prognostic potential and therefore might be used as a clinical biomarker [14]. Indeed, for patients with PCa it was shown that an increase in serum GDF-15 levels was associated with metastatic castration-resistant PCa and rapid disease progression [2].

In the current retrospective analysis, the role of serum hepcidin and GDF-15 as prognostic markers in upper urinary tract urothelial carcinomas (UUTUC) and renal cell carcinoma (RCC) were investigated. Urothelial carcinomas (UCs) are malignant neoplasms of the specific epithelium of the urinary tract with UUTUC representing 5–10% of UCs [15, 16]. This type of cancer is very aggressive and has a high recurrence rate [17–19]. Despite the aggressive nephroureterectomy, which is the standard treatment of UUTUC, prognosis mainly depends on tumor stage and grade with a 5-year cause-specific survival of > 90% for Ta and T1 tumors and < 10% for T4 tumors [17, 18]. Nevertheless, reliable prognostic markers are needed to identify high-risk UUTUC patients in sufficient time to select the best treatment and follow-up strategies for each patient. Although many biomarkers had been investigated, until now, none of them was implemented in clinical practice.

A similar problem exists for the entity RCC, which is the most common type of kidney cancer that occurs in 90% of all kidney cancers. During the course of disease, up to 50% of patients will develop metastases and, if present, are likely to die [20].

Despite all efforts that have been made to identify biomarkers that might play a role in kidney cancer prognosis [21], until today no viable biomarker has been introduced into clinical practice. The success of correct risk stratification after nephrectomy depends on our ability to distinguish between high risk and low risk patients. Therefore, prognostic biomarkers are in need to individualize follow-up care and identify those patients who are likely to relapse.

In the presented study, the potential of serum hepcidin and serum GDF-15 as potential biomarkers in UUTUC were tested and reevaluated in RCC as already investigated by Kamai and colleagues [9]. Serum hepcidin and GDF-15 levels were increased in patients with metastatic RCC and UUTUC compared to non-metastasized disease. Serum hepcidin and GDF-15 levels correlated with patient’s outcome. Therefore, the data support the further assessment of serum hepcidin and GDF-15 as prognostic markers in RCC and UUTUC.

## Methods

### Study design

This retrospective study has been approved by the Ethical Committee of the University of Muenster (Study No 2014–181-f-S). All patients and controls involved in the study provided a written approval to store serum samples, measure serum hepcidin and GDF-15 level, and retrospectively analyze it in relation to patient’s medical history and demographical data. The study was performed according to the Declaration of Helsinki. Sera of patients suffering from RCC or UUTUC were used to measure serum hepcidin and serum GDF-15 levels and retrospectively analyzed in relation to patient’s medical history.

### Patient cohort

Inclusion criteria were age > 18 years, hospitalization in the Department of Urology at University Hospital Muenster, either a non-malignant cause for hospitalization (UUTUC or RCC with or without metastases) or cancer relapse between May 2010 and July 2014. All patients were hospitalized due to surgery.

The controls were not matched with the UUTUC or the RCC patients. Controls were chosen from all patients at Muenster University Hospital without cancer undergoing surgery. Table 1 depicts patient’s characteristics. In total, *n* = 38 patients were diagnosed with UUTUC: *n* = 7 patients presented with metastases and *n* = 17 patients presented with relapse. For RCC, *n* = 94 patients were chosen, out of which *n* = 44 patients presented with metastases, who were classified according to the Memorial Sloan Kettering Cancer Center (MSKCC) Risk Score, and *n* = 39 patients were diagnosed with relapse.

**Table 1:** Summary of patients’ characteristics

	Urothelial Carcinoma		Renal Cell Carcinoma		Control
	Metastases-free	Metastases	Metastases-free	Metastases	
Number of Patients	31 (♂ 22, ♀ 9)	7 (♂ 4, ♀ 3)	50 (♂ 35, ♀ 15)	44 (♂ 30, ♀ 14)	21 (♂ 15, ♀6)
Age	69 [57.5, 73.5]	71 [70.5, 71.5]	65.2 [56.9, 73.2]	63.1 [55.3, 69.2]	66 [61, 73]
TNM classification	CIS, Ta, T1–3 N0, M0	Ta, T1–3 N+/M+	T1a-3c N0–1 M0	T1a-4 N0–2 M0–2	–
Hgb (g/dl)	F: 12.9 [10.9, 13.9] M: 13.8 [13.2, 14.4]	F: 10.7 [10.6, 11.9] M: 13.8 [12.5, 14.3]	F: 13.3 [12.8, 14.4] M: 14.5 [12.8, 15.5]	F: 11.6 [10.7, 12.9] M: 14.2 [12.6, 14.7]	F: 13.6 [12.9, 14.2] M: 14.9 [14.5, 15.9]
Hepcidin (ng/ml)	36.4 [19.1, 113.3]	59.6 [46.7, 110.8]	8.2 [4, 18.4]	18 [8.3, 35.3]	5.8 [3.9, 11.2]
GDF-15 (pg/ml)	2039.4 [1360.9, 3215.2]	4342.3 [3448, 6406.6]	934.6 [687.1, 1711.2]	1742.6 [989.1, 2690.1]	754.3 [601.3, 987.7]
Relapse	12	5	0	39	–

In addition, *n* = 21 patients without renal or bladder disease, infection, any type of cancer or anemia were analyzed. These patients were also hospitalized at the University Hospital Muenster in the departments of orthopedics, vascular and endovascular surgery, neurosurgery, trauma surgery or cardiac or thoracic surgery.

All serum samples were taken preoperatively while patients were either in the hospital for a preoperative surgical and anesthesia visit several days prior to hospital admission or they were already hospitalized at the University Hospital Muenster. Serum samples were stored at − 80 °C until use.

### Patient’s data

The following parameters of patients’ data were retrospectively collected from electronic data files and used for the analyses: gender, date of birth, date of diagnosis, hospitalization date, classification and tumor stage, MSKCC Risk Score (only for RCC), preoperative hemoglobin (Hgb), anemia, date of last contact or, if applicable, date of death, occurrence of metastases at the time of the blood collection and cancer relapse. Relapse is defined as cancer at the same site after a cancer free interval. The expression recessive is used equivalent to relapse. Patients with “metastases” already had metastases at the time of the blood collection. Overall survival was calculated as time span between date of blood draw and death. Patients who were still alive at the time of the last contact are included as right-censored. Electronic files were checked until May 2015 for UUTUC and July 2016 for RCC.

### ELISA measurements of serum hepcidin and GDF15

Serum hepcidin levels were measured in duplicates with the Hepcidin 25 (bioactive) HS ELISA (Cat. No. EIA-5782, DRG) assay according to the manufacturer’s instructions. Serum GDF-15 levels were measured with the Quantikine ELISA Human GDF-15 Immunoassay (Cat. No. DGD150, R&D Systems) according to the manufacturer’s instructions. Absorbance was measured at 450 nm using a BioTek photometer (EL808, BIOTEK® Instruments INC.) and analyzed with the software Gen5 (Gen5 Data Analysis Software, BIOTEK® Instruments INC.).

### Statistical analyses

All statistical analyses were carried out using GraphPad Prism 6® (La Jolla, CA, USA). Data are expressed as median [Q1,Q3]. Serum hepcidin and serum GDF15 levels were analyzed using non-parametric Mann-Whitney-U tests with a two-tailed *p*-value for the comparison of two groups, and Kruskal-Wallis tests with Dunn’s test for pairwise comparisons for the comparison of more than two groups. Correlations were analyzed using non-parametric Spearman correlation with a two-tailed p-value. Multivariable analyses were performed to adjust for age and gender in non-matched patient cohorts.

For Kaplan-Meier overall survival analysis, patients were divided into two groups depending on their hepcidin and GDF-15 level as performed previously by Kamai et al. (9) and Wollert et al. [22], respectively. Survival curves were compared using the log rank test. All inferential statistics are intended to be exploratory (hypothesis generating), not confirmatory, and are interpreted accordingly. Therefore, no adjustment for multiple testing was applied.

## Results

*Patients’ characteristics -* Patient’s characteristics are shown in Table 1. In total, data of 38 patients suffering from UUTUC were analyzed. The metastases-free group consisted of *n* = 31 patients and had an average age of 69 [57.5, 73.5] years. Tumors were TNM classified as Ta/CIS/T1–3 N0 M0. The metastatic group (lymphnode or distant metastasis) included *n* = 7 patients with an average age of 71 [70.5, 71.5] and TNM stages were Ta/T1–3 N+ M+. In total, 17 patients with UUTUC experienced cancer recurrence. In female patients, the Hgb level was similar to the Hgb level in control patients. Male patients with metastases (Hgb 13.8 [12.5, 14.3] g/dl; *p* = 0.03) and without metastases (Hgb 13.8 [13.2, 14.4] g/dl; *p* ≤ 0.0001) had a noticeable lower Hgb level compared to male control patients (14.9 [14.9, 15.9] g/dl).

For RCC, data of 94 patients were analyzed; *n* = 50 patients were metastases-free and without cancer recurrence. 44 patients presented with metastases, 39 of these 44 experienced cancer relapse. The average age was 65.2 [56.9, 73.2] years for the metastases-free group and 63.1 [55.3, 69.2] years for patients with metastases. Metastases-free patients had a TNM classification of T1a-3 N0–1 M0, while the tumors of patients with metastases were classified T1a-4 N0–2 M0–2. The preoperative Hgb levels were decreased in the metastatic group compared to controls for both, male and female patients. While male patients with metastases had Hgb levels of 14.2 [12.6, 14.7] g/dl, the Hgb levels in male control patients were 14.9 [14.5, 15.9] g/dl (*p* = 0.001). Female patients with metastases had Hgb levels of 11.6 [10.7, 12.9] g/dl, while control patients presented with Hgb levels of 13.6 [12.9, 14.2] g/dl (*p* = 0.02).

**Fig. 1:**
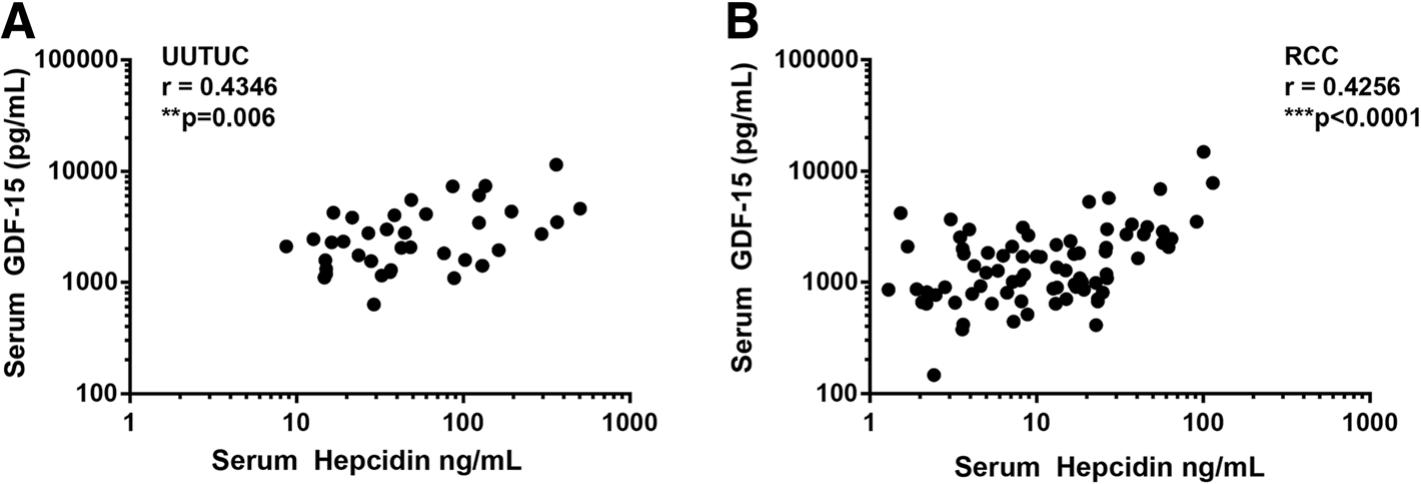
Positive correlation of serum hepcidin and GDF-15 levels in both, UUTUC and RCC patients. Correlation analysis of serum hepcidin and GDF-15 levels revealed a positive correlation for both patient cohorts, **(a)** UUTUC (*r* = 0.4346, **p = 0.006) and **(b)** RCC (*r* = 0.4256, ***p < 0.0001)

Serum hepcidin and GDF-15 levels in patients suffering from UUTUC (Fig. 1a, *r* = 0.4346; *p* = 0.006) or RCC (Fig. 1b, r = 0.4256; *p* < 0.0001) were positively correlated.

**Fig. 2:**
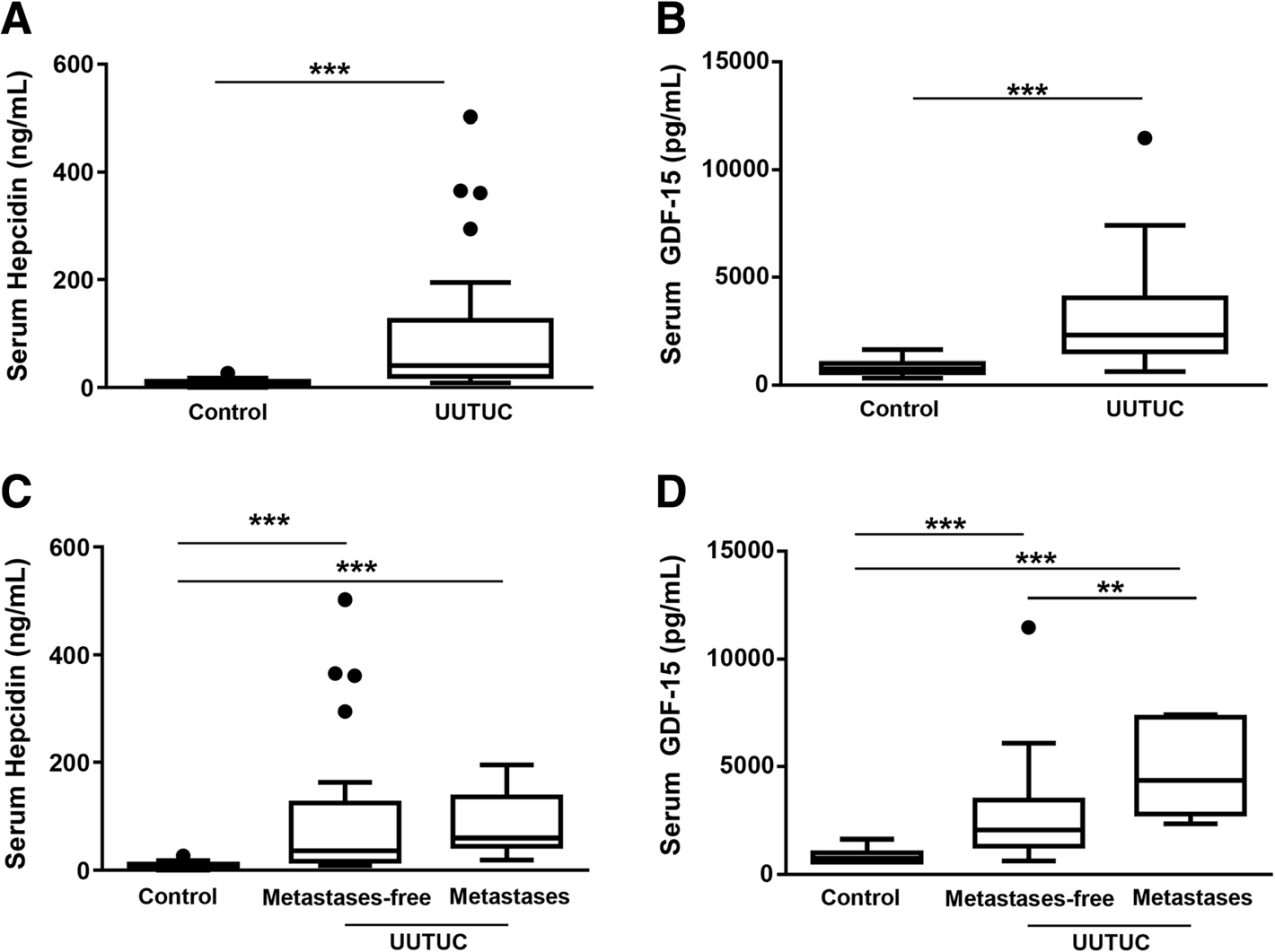
Increased serum hepcidin and GDF-15 levels in patients suffering from UUTUC compared to control patients. Serum hepcidin and GDF-15 levels from patients with UUTUC were analyzed and compared to patients without cancer or infection. Patients with UUTUC presented with increased **(a)** serum hepcidin (***p ≤ 0.0001) and **(b)** serum GDF-15 (***p ≤ 0.0001) levels compared to controls. **(c)** Serum hepcidin levels (***p ≤ 0.0001) and **(d)** serum GDF-15 levels are shown (***p ≤ 0.0001; **p = 0.003) of controls and the patient cohort divided into the two subgroups of patients with and without metastases

*Serum hepcidin and GDF-15 levels in patients suffering from UUTUC -* In order to test whether hepcidin and GDF-15 were possible prognostic markers for the prognosis and classification as well as the prediction of patients’ outcome for the entity UUTUC, serum hepcidin and GDF-15 levels were analyzed in patients’ sera with and without UUTUC. Patients with UUTUC had higher serum hepcidin levels (40.6 [22.1, 118.8] ng/mL vs. 5.8 [3.9, 11.2] ng/mL; *p* ≤ 0.0001, Fig. 2a) as well as higher serum GDF-15 levels (2303.7 [1560.9, 3968] pg/mL vs. 754.33 [601.3, 987.7] pg/mL; p ≤ 0.0001, Fig. 2b) compared to control patients. To analyze the role of serum hepcidin and GDF-15 and its association with the severity of the disease, patients were divided into two subgroups of patients with or without metastases. When compared to control patients, the non-metastatic as well as the metastatic subgroup had higher serum hepcidin as well as serum GDF-15 levels (*p* ≤ 0.0001, Fig. 2c and d). Interestingly, the non- metastatic subgroup hat lower serum GDF-15 levels when compared to the metastatic group (*p* = 0.003, Fig. 2d).

**Fig. 3:**
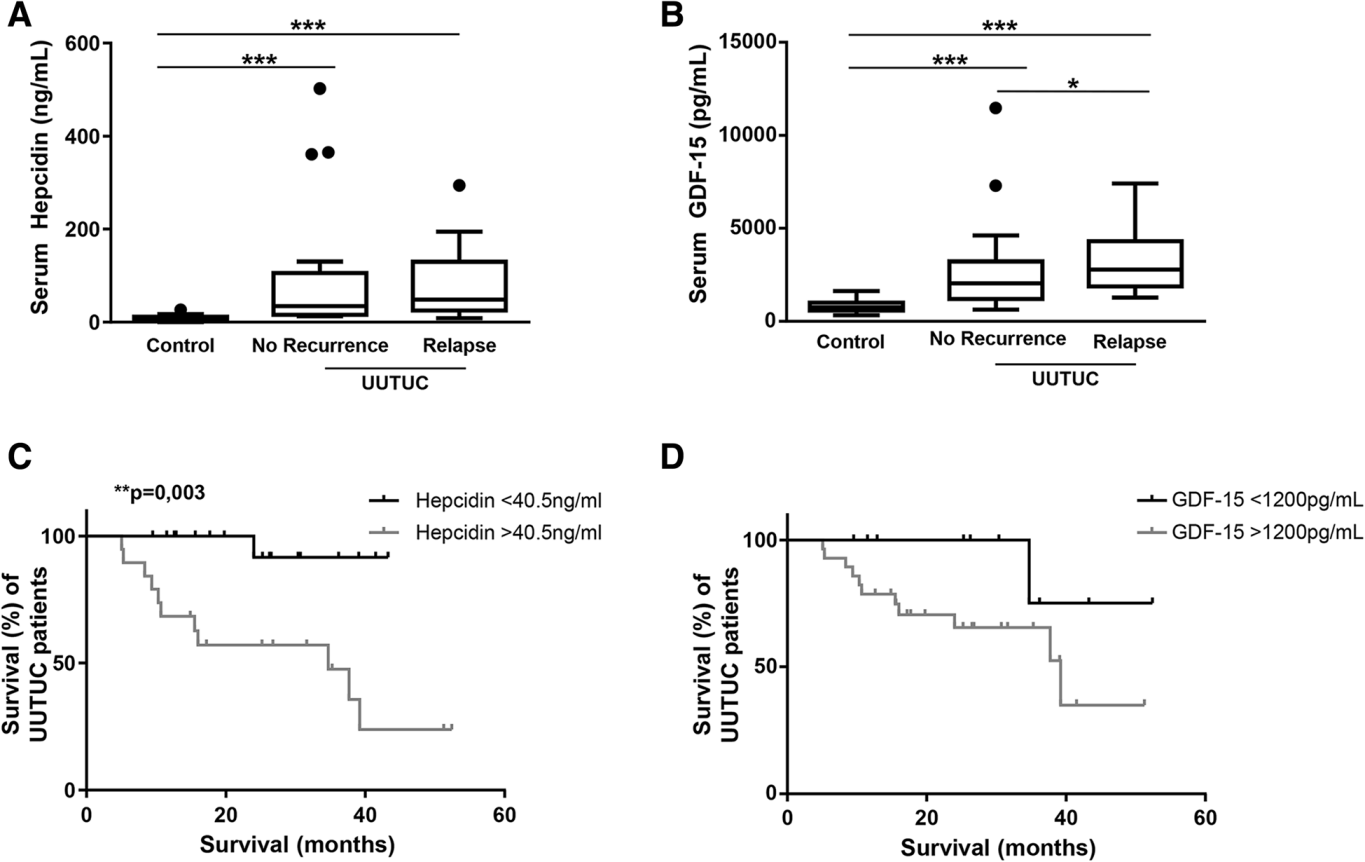
Increased hepcidin and GDF-15 serum levels were associated with patient’s outcome in UUTUC. For analysis of relapse patients were divided into the two subgroups of (a) patients with relapse and (b) patients without relapse. **(a)** Serum hepcidin levels (***p ≤ 0.0001) and **(b)** serum GDF-15 levels (***p ≤ 0.0001, *p = 0.03) of UUTUC patients were compared to control patients. **(c)** For survival analysis patients were assigned to two groups according to their serum hepcidin and GDF-15 levels, respectively. Patients with serum hepcidin levels below 40.5 ng/ml had a higher survival than patients with serum hepcidin levels above 40.5 ng/ml (Log-Rank **p = 0.003). Ticks indicate right-censoring. **(d)** There was a trend towards higher survival for patients with GDF-15 serum levels above 1200 pg/ml (Log-Rank *p* = 0.076)

Next, an association of relapse and patients’ outcome with an increase of serum hepcidin and GDF-15 was investigated. For relapse analyses, patients were divided into two subgroups of patients with or without relapse, and were compared to controls. Patients suffering from UUTUC presented with higher hepcidin serum levels in the relapse (48.76 [23, 124.3] ng/mL) as well as in the non-recurrent subgroup (34.76 [16.3, 88] ng/mL) compared to controls (5.8 [3.9, 11.2] ng/mL; both p ≤ 0.0001; Fig. 3a).

Also, GDF-15 serum levels were increased in the relapse (4342.29 [1939.4, 4253.7] pg/mL) as well as in the non-recurrent subgroup (2039.43 [1230.9, 2999.4] pg/mL) when compared to controls (754.3 [601.3, 987.7] pg/mL; both p ≤ 0.0001). Serum GDF-15 in the relapse subgroup was statistically noticeable compared to the non-recurrent subgroup (*p* = 0.03; Fig. 3b).

Next, the overall survival of patients with a serum hepcidin level higher than the overall median was compared to survival of patients with a serum hepcidin level up to the median. For UUTUC, the overall median of serum hepcidin was 40.5 ng/ml. As shown in Fig. 3c survival of UUTUC patients with hepcidin levels above 40.5 ng/ml was lower than survival of patients with hepcidin levels below 40.5 ng/ml (*p* = 0.003). There was a notion towards decreased survival (in months) in patients with serum GDF-15 levels above 1200 pg/ml compared to survival of patients with serum GDF-15 levels below 1200 pg/ml (*p* = 0.076; Fig. 3d).

In summary, high serum hepcidin and GDF-15 levels were associated with metastases, relapse as well as a decrease in survival. Therefore, the results suggest that serum hepcidin and serum GDF-15 levels might be reliable prognostic markers in UUTUC*.*

*Serum hepcidin and GDF15 levels in patients suffering from RCC-* Kamai et al. have postulated that serum hepcidin levels may be used as a prognostic marker in RCC. In the current paper, the possibility of hepcidin as a prognostic marker in RCC was re-evaluated using a larger cohort of patients (94 patients vs. 32 patients). Furthermore, the use of GDF-15 serum levels as a second prognostic marker was investigated.

**Fig. 4:**
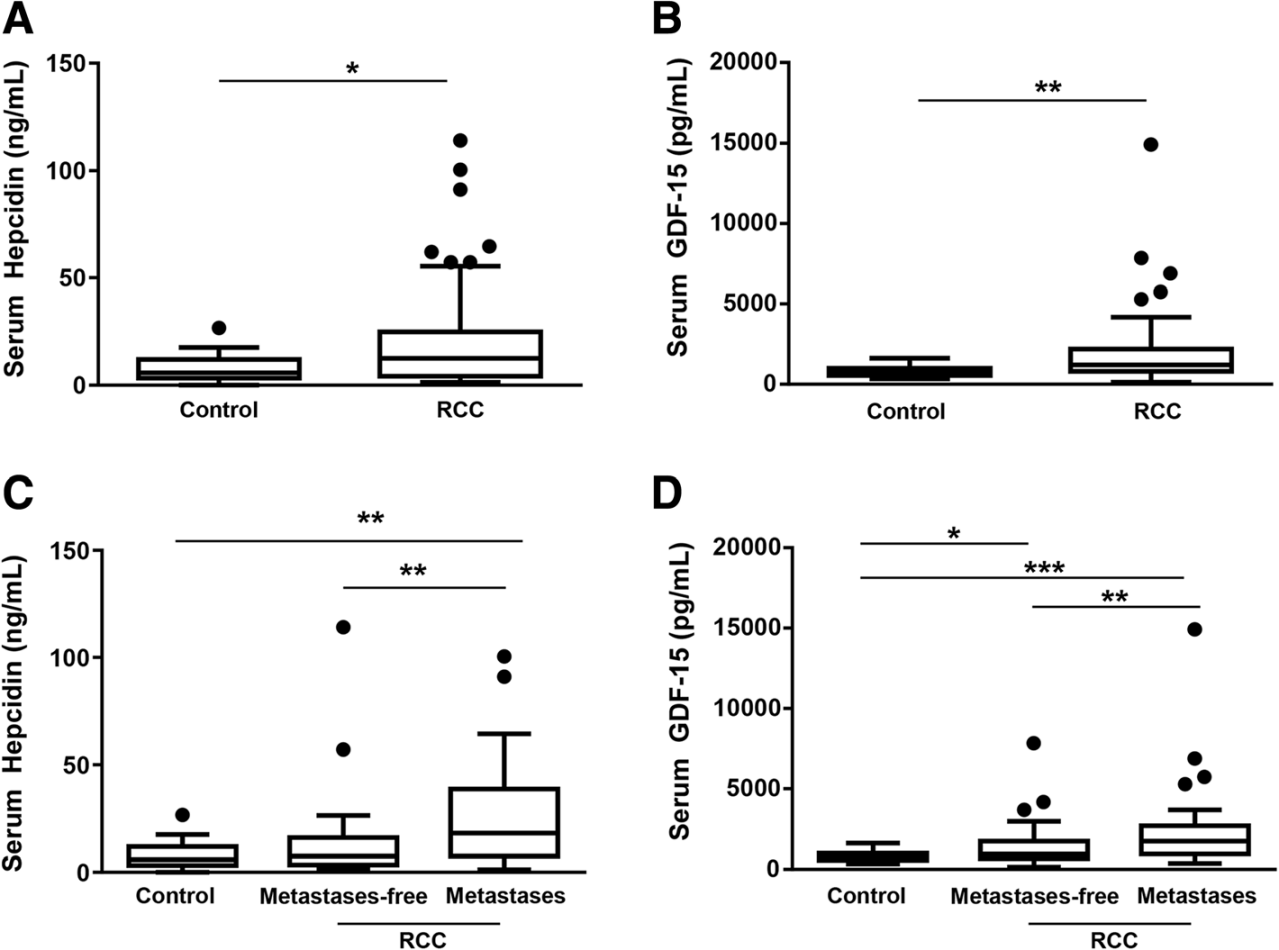
Higher serum hepcidin and GDF-15 levels in patients with renal cell carcinoma (RCC) compared to patients without cancer or infection. Serum of patients with RCC was analyzed for hepcidin and GDF-15 levels and compared to controls. Patients with RCC presented with increased **(a)** serum hepcidin (*p = 0.03) and **(b)** serum GDF-15 (**P = 0.0003) levels compared to controls. **(c)** The cohort was divided into the two subgroups of patients with and without metastases. Serum hepcidin levels are shown (**p ≤ 0.004). **(d)** Serum GDF-15 levels are depicted (***p ≤ 0.0001; **p ≤ 0.02)

In patients suffering from RCC, serum hepcidin (12.55 [4.4, 24.1] ng/mL vs. 5.82 [3.9, 11.2] ng/mL, *p* = 0.03, Fig. 4a) as well as GDF-15 (1215 [807.8, 2151.7] pg/mL vs. 754.33 [601.3, 987.7] pg/mL, *p* = 0.0003, Fig. 4b) levels were significantly increased compared to control patients. As described above, patients were divided into the two subgroups of patients with and without metastases. This classification revealed that patients suffering from metastases had higher hepcidin serum levels compared to controls (*p* = 0.001) and metastases-free patients (*p* = 0.004, Fig. 4c). An increase in GDF-15 serum levels was observed in the metastases-free (*p* = 0.024) as well as in the patients with metastases (*p* ≤ 0.0001) group when compared to controls, with even higher GDF-15 serum levels in the metastases group (*p* = 0.002, Fig. 4d).

**Fig. 5:**
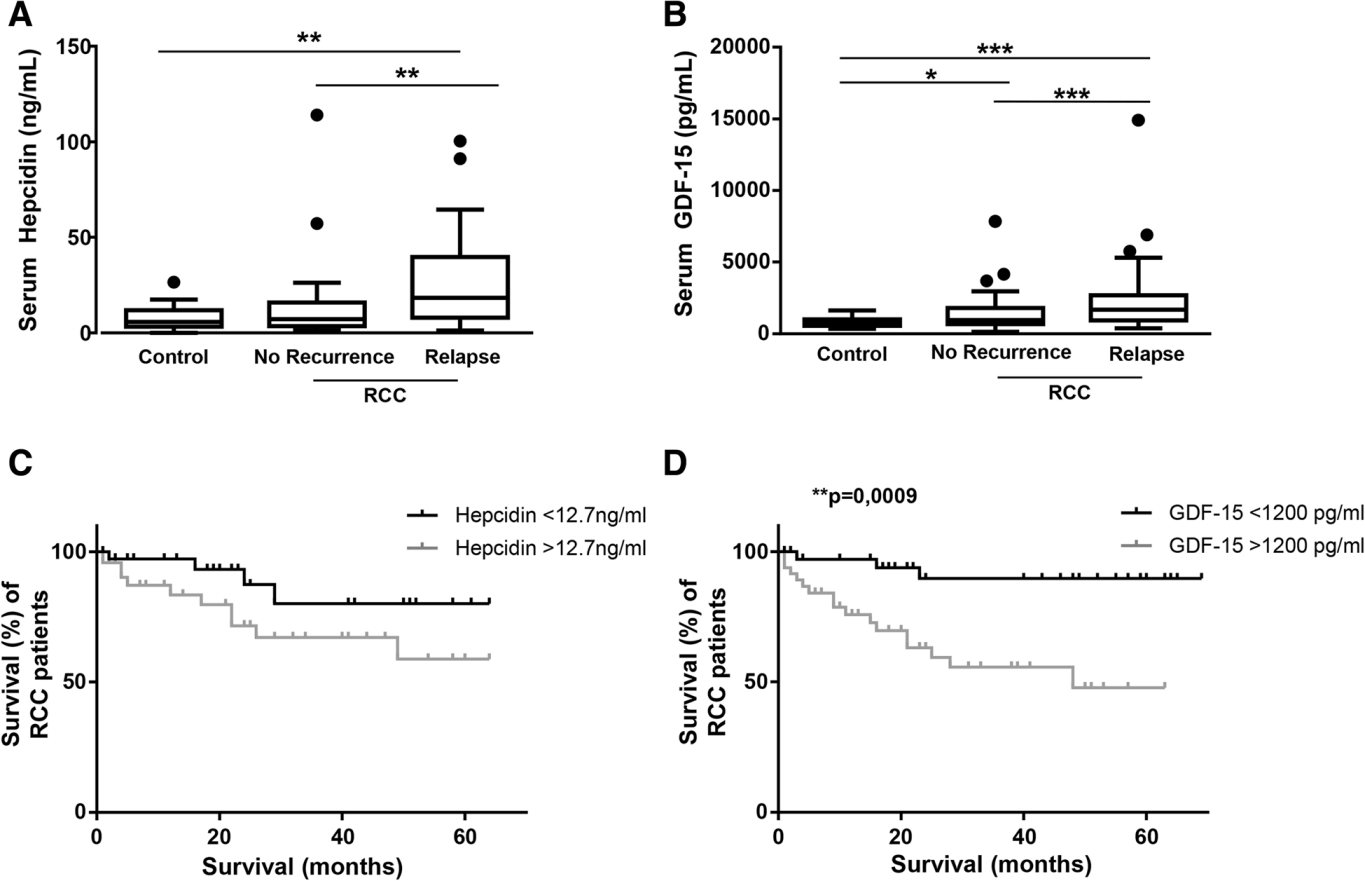
Patients’ outcome in RCC was associated with serum hepcidin and GDF-15 levels. For relapse analysis, patients were divided into the two subgroups of patients with or without relapse. **(a)** Serum hepcidin (**p ≤ 0.002) and **(b)** serum GDF-15 (***p ≤ 0.0001, *p = 0.02) levels of RCC patients were compared to control patients. **(c)** For survival analysis patients were assigned to two groups according to their serum hepcidin and serum GDF-15 levels, respectively. Patients with serum hepcidin levels below 12.7 ng/ml had a trend towards higher survival than patients with serum hepcidin levels above 12.7 ng/ml (Log-Rank *p* = 0.09). Ticks indicate right-censoring. **(d)** Patients with GDF-15 serum levels below 1200 pg/ml had a higher survival than patients with GDF-15 serum levels above 1200 pg/ml (Log-Rank **p = 0.001)

To analyze whether patients’ outcome was associated with an increase of serum hepcidin and GDF-15, the cohort was classified into the two subgroups with and without relapse. In patients with RCC, hepcidin levels were higher in the relapse subgroup (18.39 [8.2, 28] ng/mL) compared to both, control patients (5.82 [3.9, 11.2] ng/mL; *p* = 0.001) and the recurrence-free subgroup (7.13 [3.6, 15.5] ng/ml; p = 0.002, Fig. 5a). There was no noticeable difference between controls and patients without recurrence. Serum GDF-15 levels were significantly increased in both, non-recurrent (934 [691.2, 1762.9] pg/mL; p = 0.02) and the relapse subgroup (1742 [973.4, 2690.9] pg/mL; *p* ≤ 0.0001) compared to controls (754.3 [601.3, 987.7] pg/mL). Moreover, the increase in GDF-15 serum levels in the relapse subgroup was higher compared to the no-recurrence subgroup (Fig. 5b).

Analyzing the correlation of overall survival of patients and serum hepcidin and GDF-15 levels revealed a trend towards a decreased survival of patients with serum hepcidin levels higher than 12.7 ng/mL (*p* = 0.09; Fig. 5c).

Furthermore, patients with serum GDF-15 levels higher than 1200 pg/ml presented with a decrease in survival (*p* = 0.001; Fig. 5d).

Additionally, we performed multivariable analysis to adjust for age and gender in non-matched patient cohorts. The multivariable analysis confirmed the findings of the study.

The results suggest that high hepcidin and GDF-15 serum levels were associated with metastatic RCC as well as the incidence of relapse and, at least for GDF-15 serum levels, with a decrease in survival. Therefore, as also suggested for UUTUC, serum hepcidin and GDF-15 might be evaluated as possible prognostic markers in RCC.

## Discussion

Prognostic biomarkers for aggressive cancer entities are highly relevant to monitor therapy, predict efficacy of treatment and thereby guide therapeutic decisions and to predict patients’ survival. In the current study the serum levels of the two hormones serum GDF-15 and hepcidin were analyzed with regard to their possible prognostic properties to determine severity of the disease and survival of patients suffering from either UUTUC or RCC. The results show that metastases and relapse in both entities was accompanied by high serum hepcidin and GDF-15 levels. A correlation analysis of hepcidin/GDF15 and hemoglobin levels revealed that hepcidin and GDF15 correlated with hemoglobin levels in cancer patients, but not in controls. This correlation was expected, as cancer is known to induce hepcidin. Hepcidin, in turn, causes iron deficiency and provokes anemia by impeding the iron absorption channels ferroportin to absorb iron from the intestine and to release iron from iron storage cells such as macrophages**.** The overall survival was decreased in patients with hepcidin and GDF-15 serum levels that were above the overall median. Of note, we know that patients with recessive die earlier. We also know now that patients with high serum hepcidin levels die earlier. This leads to the hypothesis that high serum hepcidin levels might have a prognostic value. Within the cohort of patients with high serum hepcidin levels, we did not analyze the prognostic value of hepcidin, as the number of patients included in this study was small. In a future study, we aim to determine hepcidin at different time points within the disease progression to evaluate the prognostic potential of hepcidin. Nevertheless, the data suggest that hepcidin and GDF-15 might be possible prognostic marker in the entities UUTUC and RCC to determine the severity of the dieses as well as patient’s outcome.

In this study *n* = 38 patients suffering from UUTUC and *n* = 94 patients with RCC were included and analyzed retrospectively. Despite the small cohort that was included in this study, particularly in cases of UUTUC, the results show statistically noticeable differences in serum hepcidin and GDF-15 levels in controls compared to diseased patients. Retrospective analyses of larger cohorts or, if feasible prospective studies, are of course desirable to verify the suggested role of the mentioned hormones as prognostic markers. As the incidence of an UUTUC is rare, collection of patient’s samples is difficult and requires time. Nevertheless, the differences in GDF-15 and hepcidin serum levels in patients with metastases and relapse justify further multi-cohort studies to implement both hormones as prognostic biomarkers in clinical practice.

Hepcidin is not only known as the regulator of iron homeostasis, but also as a prognostic marker in cancer entities: an increase in hepcidin expression was linked to patient’s outcome in prostate cancer, breast cancer, non-Hodgkin lymphoma and RCC [1–3, 9–12]. In patients with malignant breast cancer hepcidin serum levels were higher than in control patients [12]. Pinnix et al. have shown that human breast cancer tissue expresses hepcidin and ferroportin, the sole iron export channel in vertebrates: low ferroportin gene expression was associated with poor outcome, while a combination of high ferroportin and low hepcidin was associated with a good prognosis suggesting ferroportin may be a strong and independent predictor of prognosis in breast cancer [1]. In prostatic cancer an increase in serum hepcidin levels by 25 ng/ml predicted an increase in mortality by 10% [2]. Interestingly, human prostatic cancer cells produce hepcidin and have, as already described for breast cancer, a functional hepcidin/ferroportin regulatory axis, which contributes to prostate cancer survival [3]. In patients with colorectal cancer the expression of hepcidin in cancer tissue was related to T stage, but not to metastases [23]. In RCC, Kamai and colleagues have investigated hepcidin in a small cohort of patients suffering from RCC. The results are in line with the results obtained here: increased serum hepcidin levels were linked to metastasis of renal cell carcinoma [9].

In contrast to these studies, Jakszyn et al. investigated an inverse association between gastric cancer and serum hepcidin levels: higher hepcidin levels correlated with lower cancer risk [24]. To summarize, the majority of studies suggested a negative correlation between serum hepcidin levels and survival.

The next interesting question is how hepcidin might pathophysiologically contribute to an impairment of the patient’s prognosis. To date it is unclear, if cancer cells themselves might produce hepcidin. Thereby, a hepcidin producing cancer would lead to an enhanced retention of iron in the iron storage cells and aggravate anemia. Hepcidin itself is known also as an antimicrobial agent, so that increased hepcidin production reflects a more marked immune response [25].

In mice, overexpression of ferroportin in tumor cells themselves was shown to impede the tumor growth and metastasis by compromising epithelial-mesenchymal-transition [26]. An increase in serum hepcidin decreases the cell surface expression of ferroportin and might favor tumor growth and metastases, which leads to decreased survival. Furthermore, increased hepcidin levels lead to enhanced retention of iron in iron storage cells and aggravate anemia, which per se increases morbidity and mortality**.**

The hormone GDF-15 has been reported to be overexpressed in various types of cancer including prostate, breast and ovarian cancer [14, 27, 28].

It has been suggested that GDF-15 suppresses immune cells such as macrophages by suppressing the release of certain cytokines and enhances cancer cell growth [14]. Also, increased serum GDF-15 levels were reported to be associated with metastatic prostate, colorectal, gastric, hepatocellular and breast carcinomas [2, 27–30]. In patients with metastatic melanoma and colorectal cancer higher serum GDF-15 levels were strongly associated with reduced overall survival [31, 32]. In addition, in patients diagnosed with prostate cancer, breast cancer, lung cancer and colorectal cancer higher levels of GDF-15 were associated with the occurrence of bone metastases [33]. Interestingly, in patients with ovarian cancer serum levels of GDF-15 levels were not higher in the patients with malignant neoplasms [34].

Monteiro-Reis et al. proposed that increased serum GDF-15 might be a possible prognostic marker in UUTUC to discriminate between favorable and poor outcome. These results are in accordance with the results obtained in this study: high GDF-15 levels in patients suffering from UUTUC and RCC were associated with metastases, relapse and poor survival. Therefore, we conclude that - at least for common tumors of the genitourinary tract – serum GDF-15 is a promising prognostic biomarker.

In addition, other prognostic markers are currently under investigation. For example, in patients undergoing transurethral resection of non-muscle-invasive bladder tumors the well-studied biomarker neutrophil-to-lymphocyte ratio is associated with an increased risk of recurrence, progression and worse prognosis. As a result, neutrophil-to-lymphocyte ratio could be used to improve therapeutic decisions [35, 36].

## Conclusion

To summarize, in the current study increased serum levels of hepcidin and GDF-15 were linked to metastases, relapse as well as patient’s outcome in UUTUC and RCC. Both hormones might be potential biomarkers in the entities UUTUC and RCC. Therefore, the data support the further assessment of serum hepcidin and GDF-15 as prognostic markers in RCC and UUTUC.

## Abbreviations

ACD: Anemia of chronic disease
AI: Anemia of inflammation
GDF-15: Growth/differentiation factor 15
PCa: Prostate cancer
RCC: Renal cell carcinoma
UC: Urothelial carcinoma
UUTUC: Upper urinary tract urothelial carcinomas
